# MaskMyPy: python tools for performing and analyzing geographic masks

**DOI:** 10.1186/s12942-025-00399-6

**Published:** 2025-05-09

**Authors:** David Swanlund, Nadine Schuurman

**Affiliations:** Department of Geography, Simon Fraser University, 8888 University Drive, Burnaby, BC V5A 1S6 Canada

**Keywords:** Geographic masking, Geomasking, Geoprivacy, Privacy, Python, Anonymization, Tools

## Abstract

**Background:**

Geographic masking is an important but under-utilized technique for protecting and disseminating sensitive geospatial health data. Geographic masks work by displacing static point locations such that the people those locations describe cannot be identified, while at the same time preserving important spatial patterns for analysis. Unfortunately, there is a lack of available tooling surrounding geographic masks which we believe creates an unnecessary barrier towards the adoption of these techniques. As such, this article presents a set of tools for performing, evaluating, and developing geographic masks, called MaskMyPy.

**Results:**

MaskMyPy is an open-source Python package that includes functions for performing geographic masks, including donut, street, location swapping, and Voronoi masks. It also includes a range of tools for evaluating the results of these masks, both with regard to privacy and information loss. Finally, it includes a special class called the ‘Atlas’ that aims to dramatically streamline mask execution and evaluation. We conducted a short case study to illustrate the power of MaskMyPy in geographic masking research, and in doing so showed that mask performance can range widely due solely to randomization. As such, we recommend that masking researchers test their masks repeatedly across a variety of test datasets.

**Conclusion:**

MaskMyPy makes it easy to apply a variety of geographic masks to a set of sensitive points and then measure which mask provided the most privacy while suffering the least information loss. We believe this style of tooling is important to not only make geographic masks accessible to non-experts, but to enable expert users to better interrogate the masks they develop, and in doing so drive the geographic masking discipline forward.

## Background

Whether through the use of spatial statistics or cartographic visualization, geospatial data can be a powerful resource for researchers looking to link health outcomes to environmental risk factors. As important as this type of research is, however, it cannot come at the expense of patient privacy; as the saying goes, ‘first, do no harm’. Geographic masks are techniques that offer an avenue for exploring and distributing useful spatial health data without violating the privacy of the people that data describes. Sadly, geographic masks are not readily available to the users that would benefit most from them. Major GIS applications and libraries alike lack geographic masking functions, requiring would-be users to dive into niches of academic literature to identify, understand, implement, and evaluate the masks that would benefit them. For many, this friction simply puts geographic masks out of reach. This can lead to one of two problems: either sensitive data gets published without being properly anonymized, violating privacy, or it stays locked away, preventing other researchers from being able to explore it whatsoever [1, 2]. The goal of this article is to showcase geographic mask tooling that makes both of these outcomes more easily avoidable.

Fundamentally, geographic masks transform spatial point data with the intent to protect geoprivacy while simultaneously preserving the utility of that data for analysis [3]. Unfortunately, these two objectives are fundamentally at odds: the more we do to protect privacy in a spatial dataset, the less useful the data becomes. The goal of any geographic mask is to optimize this trade-off, such that privacy is sufficiently protected while incurring the least amount of information loss. Many geographic masks have been developed over the years, each attempting in their own way to best satisfy this fundamental goal [4–6]. As a result, there are now a variety of masks to choose from. Actually making this selection, however, can be quite difficult.

This difficulty stems from the fact that every mask performs differently depending on the underlying nature of the data, and measuring these differences can be the basis of an entire research publication by itself. Many geographic masking studies use a wide variety of methods for assessing privacy protection and information loss, often with interpretations that are at odds with each other [7]. Moreover, even if a would-be masker found three studies that each used identical methods to assess a handful of different masks, their results would be difficult to utilise as they are specific to the dataset being masked. For instance, variations in population density and urban form can greatly affect the outcome of a masking procedure [8]. A mask applied to data in a sprawling city like Orlando would likely perform quite differently than in a highly dense city like New York.

The underlying structure of the data itself also affects geographic masking: how many points are there, are they dense or sparse, who or what are they describing, are they clustered, do they span extremely mixed population densities, and do they represent fixed locations or do they move over time? Further complicating things is the fact that most masks have parameters, such as maximum displacement distance, that must be taken into account. It is impossible to know what the optimal parameters are without first testing a variety of them. Finally, mask performance can vary solely due to the randomization element that many masks rely on, meaning that studies that evaluate masks should ideally do so over many iterations of the mask. Ultimately, each of these factors add up such that just because one study showed a given masking technique to be the best for *their* data and context, does not mean it will be the best for *your* data and context.

This means that it is important to perform some level of testing and validation on a masked dataset before publishing it. Such testing may not need to be as comprehensive as a full research study, but there should be some level of validation nonetheless. Unfortunately, this is both difficult and time consuming. Geographic masking can already be a burdensome process even without performing this analysis, and studies have shown that many researchers forgo privacy protection entirely and simply publish sensitive location data in maps [1, 2].

Our previous research has attempted to reduce this friction by developing both masks and tools that are more simple to use [9, 10]. This article is an extension of this research, and describes an open-source Python package for geographically masking point data, called MaskMyPy. MaskMyPy was first introduced within a 2020 article proposing a new method for geographic masking, called Street Masking [10]. At the time, the software largely focused on providing a small number of easy-to-use geographic masking functions for anonymizing GeoDataFrames, with the Street mask being its primary feature. This article presents a new, more powerful iteration of this tool with a much larger focus on the *analysis* aspect of geographic masking. It allows users to quickly execute a number of geographic masks along with any combination of parameters on a given dataset, all while automatically calculating privacy and information loss metrics. This allows for rapid comparison and evaluation of mask performance, making it far less burdensome to robustly protect geospatial data. Moreover, its features may help aid other researchers in developing new geographic masks, providing a framework for this research community to build upon.

This article begins with a brief overview of geographic masking and existing tools. Next, it highlights the core features of MaskMyPy, including its inbuilt masks, analysis tools, and management features. These features are then used to analyze the results of hundreds of mask iterations, with the goal of highlighting a range of often overlooked factors that impact mask performance. Finally, the article concludes by discussing the importance of, and need for, comprehensive privacy tooling in academic research.

### Geographic masks

Geographic masks have steadily evolved over the last two and a half decades. First proposed by Armstrong et al. [3], early masks are best exemplified by affine transformations and random perturbation. Affine transformations include techniques that translate, rotate, or scale point patterns globally by a predefined value in order to protect privacy. A concern with these techniques, however, is that re-identifying a small subset of the data can lead to the entire dataset being re-identified as well, as all points are transformed using the same values. A partial solution to this is to split the dataset into a grid and perform different affine transformations locally to each cell [11]. Random perturbation, on the other hand, provides a much stronger solution to this problem, as every point is displaced randomly within a given maximum distance [3]. Because each point is treated independently, re-identifying one point (or even a small subset of points) in a given dataset cannot be used to then re-identify other points in the same dataset.

Subsequent masks often tweak this basic formula in order to make up for random perturbation’s weaknesses. For instance, a weakness of random perturbation is that points may only be displaced small distances (e.g. 2 m), providing almost no privacy protection. Donut masking adds a minimum displacement distance to solve this issue [4]. Another weakness is that points can be displaced to impossible locations, such as the ocean. Location swapping and the verified neighbor mask both solve this issue by leveraging contextual address data; instead of displacing points entirely at random, they will relocate given a point to a randomly selected address nearby, helping to ensure that the masked data remains more realistic [6, 12].

However, other masks have been developed that take entirely different approaches. For instance, Adaptive Areal Elimination (AAE) seeks to provide a guaranteed minimum level of privacy [5]. It does this by iteratively aggregating census polygons until their combined population reaches a minimum threshold. Then, points are displaced within each aggregated polygon. This approach ensures that a minimum level of privacy is achieved even when the population is heterogeneously distributed.

Of course, researchers have developed more masking techniques than can be described here. Briefly, these include Street masking (which relocates points along the OpenStreetMap road network) [10], Voronoi masking (which relocates points by creating voronoi polygons and snapping them to the nearest edge) [13], multi-scale masking (which relocates points by strategically switching digits in their coordinates on the Military Grid Reference System) [14], Triangular Displacement (which relocates points based on multiple risk factors with a focus on computational efficiency) [15], NRand-K (which relocates each point based on the density of other points nearby) [16], and Adaptive Voronoi masking (which combines elements of Voronoi masking and Adaptive Areal Elimination) [17]. This is not an exhaustive list, and there are further variations of masks one may consider, such as whether to use a uniform or gaussian distribution when performing random perturbation, or whether to select the displacement distance entirely at random or by weighing it using population data. Indeed, the wide variety of ways to go about geographic masking underscores the importance of evaluation and tooling.

### Mask evaluation

Geographic masks are evaluated based on two primary concerns: how much they protect privacy, and how much information loss they incur. Beginning with privacy protection, researchers have largely settled on one primary evaluation metric: spatial k-anonymity. Spatial k-anonymity is an adaptation of the popular k-anonymity metric [18–20], which measures the uniqueness of records in a given dataset. For example, a record is 10-anonymous if it is indistinguishable from 9 other records in the same dataset. Spatial k-anonymity on the other hand may consider a masked address as 10-anonymous if there are 9 other addresses closer to it than to its original, unmasked location [21]. However, as Seidl et al. [7] note, there is a degree of disagreement in the literature regarding this definition. More specifically, should k-anonymity be measured relative to the original sensitive location, or relative to the masked location? In our opinion, and given that the goal of geographic masking is to prevent reverse geocoding of the masked data, it seems fitting to measure it based on the masked location, as this is what an adversary seeking to re-identify the address would be dealing with. Nevertheless, this does represent an inconsistency in the literature that easily goes unnoticed.

Moreover, it must be noted that spatial k-anonymity estimates are somewhat imprecise. When census data is used in the calculation, there is an inherent assumption that the population within the census area is uniformly distributed, which is rarely, if ever, the case. The use of address data ameliorates this problem, but tends to use addresses as a proxy for individuals, which introduces its own issues. Finally, spatial k-anonymity has also been measured without considering a background population, and instead considers a masked address 10-anonymous if 9 other masked addresses *within the dataset* are closer to the unmasked address than the masked address [22]. This coincides more closely with the original formulation of k-anonymity, but is extremely difficult to satisfy spatially without incurring extreme information loss.

While spatial k-anonymity poses a dominant yet somewhat ambiguous measure of privacy protection, measures of information loss are both more varied and yet more clear cut. Fundamentally, measuring information loss means measuring changes to the point pattern introduced by the masking process. This is commonly achieved by looking at how masking changes the number, location, and/or size of clusters that can be identified in the data [4, 6, 12, 19, 23]. For instance, one can use Ripley’s K function to measure clustering at multiple spatial scales on both the unmasked and masked data, and then plot the difference between the two [6, 12]. Any difference, whether it be towards greater clustering or dispersion, is an indication of information loss. Other clustering metrics that are commonly used include the average nearest neighbor index [6, 15] and SatScan [4, 12, 19, 23]. Alternatively, one can look towards changes in descriptive statistics, such as how much the mean center of the point pattern has drifted due to masking [12, 13], or by simply measuring average distance that each point was displaced [5, 12, 19]. These are only some of the many ways to quantify information loss. Ultimately, it is best to look towards multiple complementary measures to assemble a more complete picture of what is lost to the masking process.

### Existing tools

There exist a small number of tools that use geographic masks for the purpose of privacy protection, which are summarized in Table 1. In 2019 we developed MaskMy.XYZ, which is a web application for applying donut masking to sensitive locations within the browser [9]. While being very easy to use and providing rudimentary tools for measuring privacy and information loss, it is primarily designed for quickly masking small datasets rather than being a comprehensive masking application. Alternatively, GeoPriv is a QGIS plugin that comes closer to this goal, as it offers three separate masking techniques: spatial clustering, Laplacian noise, and the NRandK mask [16, 24]. GeoPriv is a plugin for QGIS, and has the advantage of being usable directly inside an existing and popular GIS environment.

**Table 1:** A summary table comparing some of the different tools available that allow users to geographically mask their data, or make use of geographic masks to achieve their desired purpose

Name	Purpose	Employed Masks	Platform	Strengths	Weaknesses
MaskMyPy	Geographic masking	Donut masking, voronoi masking, location swapping, and street masking	Python	Multiple masks, privacy metrics, and information loss metrics. Atlas feature streamlines evaluation and easily accepts custom masks	Requires users to know Python
MaskMy.XYZ [9]	Geographic masking	Donut masking	Web application	Easy to use, no installation required, includes basic privacy and information loss metrics	Cannot handle large amounts of data, single mask is relatively basic
GeoPriv [24]	Geographic masking	Spatial clustering, NrandK, Laplacian noise	QGIS	Works directly within QGIS as a plugin, includes multiple masks and an information loss metric, convenient data preview	Only one information loss metric, included masks don’t allow inclusion of geographic context (e.g. address data)
Privy.To [25]	Private location sharing	Donut masking, circle masking, political region masking	Web application	Enables users to securely share their individual location, with a range of obfuscation options	Not applicable, not intended as a geographic masking tool
MapSafe [26]	Data sovereignty	Donut masking	Web application	Enables users to notarize their spatial data on a public blockchain, no installation required, supports multiple trust levels	Not applicable, not intended as a geographic masking tool

While MaskMy.XYZ and GeoPriv are explicitly for the purpose of geographic masking, other tools make use of geographic masking to achieve a larger goal. For instance, Privy.to is another web-based application that leverages geographic masks, temporal obfuscation, and encryption to allow users to privately and securely share their location with others [25]. MapSafe takes a similar approach by combining geographic masking, encryption, and blockchain technology to allow users to anonymize sensitive geospatial data, share it based on different levels of trust, and securely notarize it on the Ethereum blockchain without exposing either the masked or unmasked data [26].

However, when compared to the wealth of geographic masking techniques that have been developed in GIScience, there is a clear lack of GISystems tooling to translate these into reality. As Boeing [27] concisely writes, “to conduct better science, we need to build better tools”. Indeed, we are in an age where spatial data abounds, but often this data is highly sensitive and cannot be easily shared without sparking legitimate privacy concerns. Translating the wealth of GIScience theory about geoprivacy and geomasking into GISystems tooling is a key step towards unlocking this data and allowing other researchers to tap into its latent potential. Better tooling can also improve the science of geographic masking itself by reducing the evaluation overhead. These are the twin goals of MaskMyPy.

## MaskMyPy: an overview

MaskMyPy was initially released in 2020 as an alpha stage software package, and included Street masking, Donut masking, as well as functions to calculate displacement distance and k-anonymity [10]. However, many lessons were learned during development about how best to implement these features in a clear and maintainable way. Moreover, the package was released as an alpha and as such uptake has remained limited. While we have had several users reach out with positive feedback, we wanted to improve the code-base to make it better to both use and contribute to, and in doing so increase uptake. This motivated us to rewrite the vast majority of the code-base to be more maintainable, readable, tested, and well packaged. From a user’s perspective, the interface was rewritten to be simpler, and many features were added to enhance the analysis aspect of geographic masking. This section outlines this new, enhanced version of the software.

MaskMyPy has three primary components: geographic masks, analysis functions, and the Atlas. This section briefly walks through using each of these components to mask a dataset and evaluate the level of privacy protection and information loss that is incurred. The goal is to demonstrate, through several short code snippets, the degree to which MaskMyPy can streamline geographic masking workflows.

### Geographic masks

**Fig. 1:**
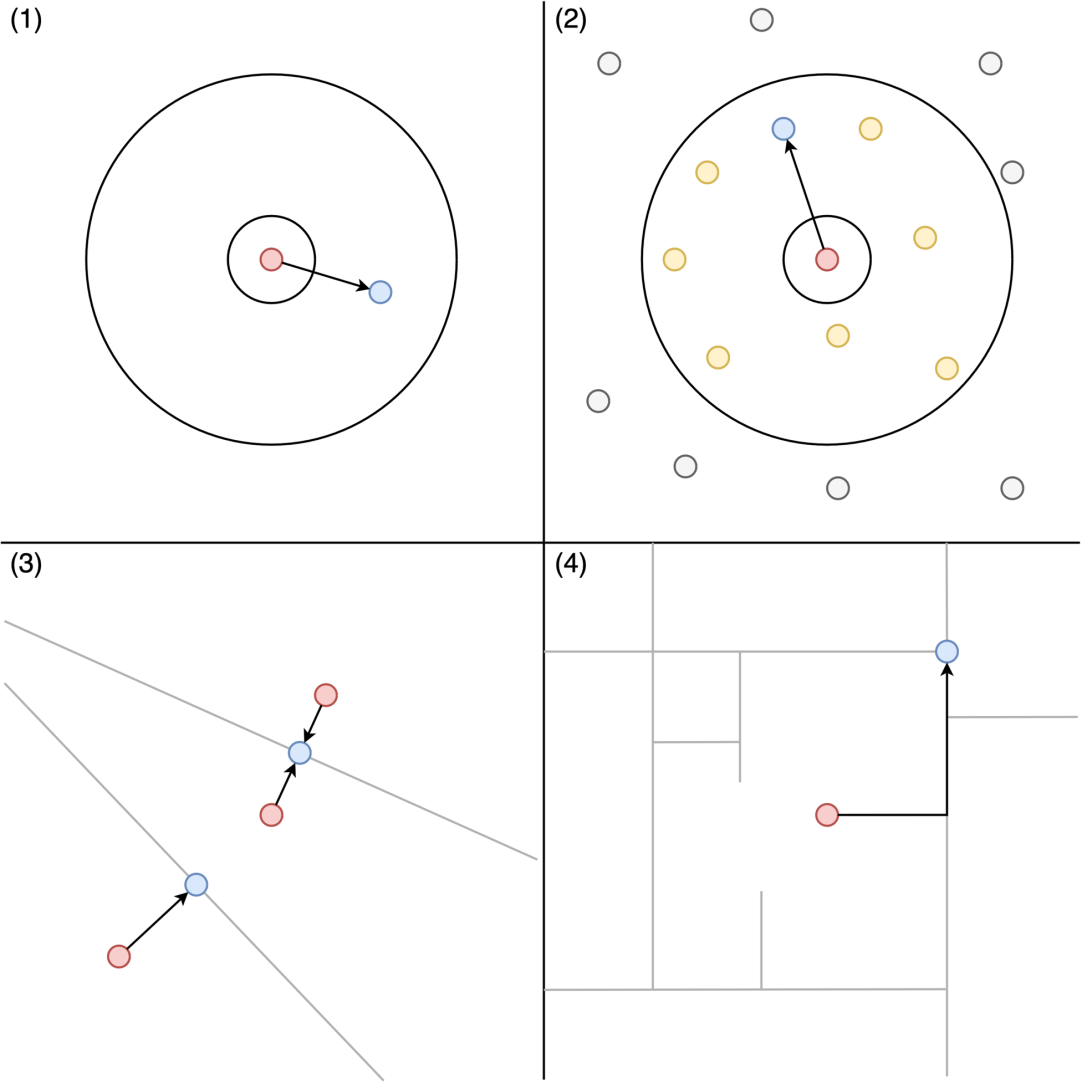
an illustration of how points are displaced by (1) donut masking, (2) location swapping, (3) voronoi masking, and 4) street masking. Donut masking (1) displaces points randomly between a minimum and maximum distance. Location swapping (2) displaces points to random nearby addresses (yellow circles) within a minimum to maximum distance. Voronoi masking (3) constructs voronoi polygons around each point, and snaps sensitive points to the closest edge (grey lines). Street masking (4) displaces points along the surrounding street network (grey lines) based on its relative density. Sensitive points are colored red, masked points are colored blue

**Fig. 2:**
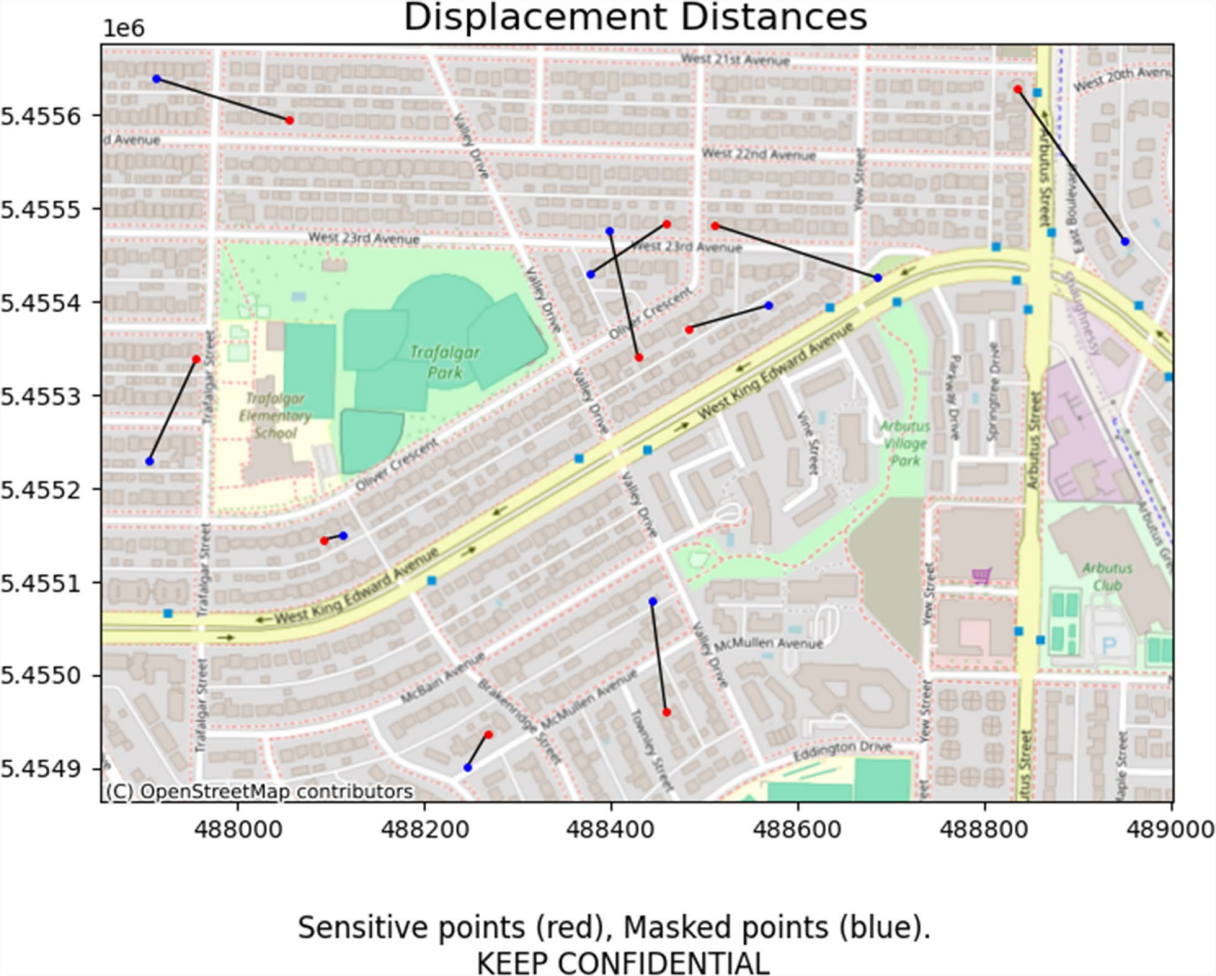
an automatically generated map depicting how each point was displaced from their original, sensitive locations (red) to the masked location (blue). Note that no actual sensitive locations were used in this example, as the data was synthetically generated


First, the included geographic masks are Python functions that take in a GeoDataFrame and various masking parameters, and return a copy of that GeoDataFrame with masked coordinates. A GeoDataFrame is a data structure that is provided by the highly popular Geopandas Python library, which provides a wide range of tools for working with spatial data. Four masks have been implemented: donut masking, location swapping, voronoi masking, and street masking. Figure 1 provides a brief explanation of how each of these masks displace sensitive points. Donut and voronoi masking provide the fastest execution times, but generally yield a worse trade-off between privacy protection and information loss than location swapping or street masking, which make use of supplemental data to better inform masking distances. As an example, the following code snippet performs donut masking, displacing points between 20 and 200 m, and producing a map (shown in Fig. 2) that visualizes how each point was displaced:

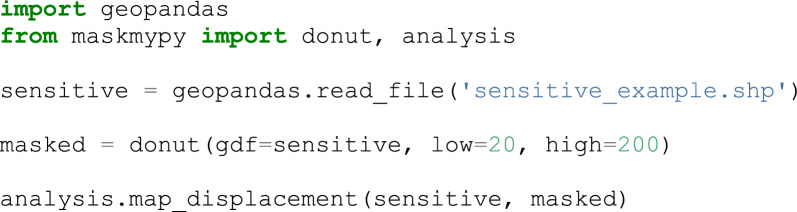


### Analysis functions

As previously mentioned, it is important to evaluate how well this mask performed, particularly with regard to privacy protection. MaskMyPy’s analysis functions drastically simplify this process. Spatial k-anonymity can be calculated using either census data (e.g. polygons with associated population counts), or address points. Building on the previous example, we will now calculate the address-based k-anonymity of our masked GeoDataFrame and then use a function that calculates the percentage of points that achieved a k-anonymity above a threshold value (in this case, 5). This technique was first used by [6], which we term here as *k-satisfaction*:
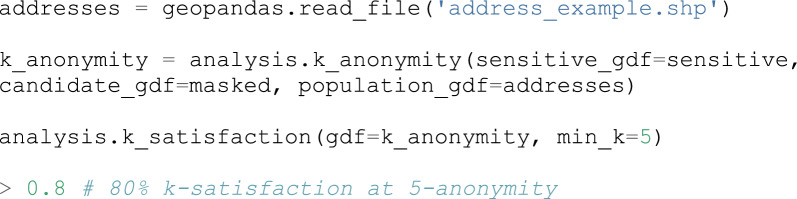


As we can see, 80% of points have achieved a k-anonymity of at least 5. If we are satisfied with this result, we can proceed to measure information loss. Several metrics are available for this purpose, including: central drift, displacement distance, nearest-neighbor distance, and Ripley’s K function. A number of higher-level functions are also available which summarize the row-level outputs of some of these metrics, facilitating interpretation. Continuing with our example, we will measure and then summarize the displacement distance of each point:
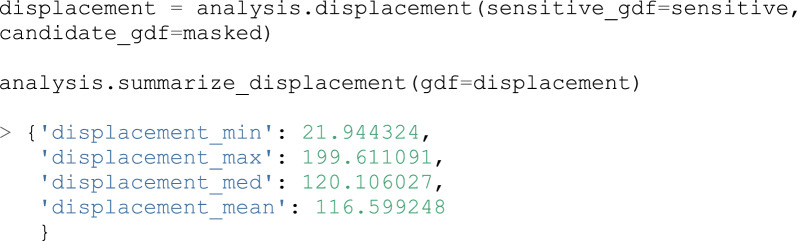


### The Atlas

While the above exemplifies some of the individual functions that MaskMyPy provides, the Atlas is a Python class that is designed to conveniently tie them together. Put briefly, it acts as a ‘mask manager’ that allows you to quickly test any number of combinations of masks and their associated parameters, automatically performing the evaluation for you and keeping track of the results. Essentially, it lets users perform a wide array of analyses in a very short amount of code, which we hope not only helps users to better validate their masks, but enables masking researchers to ask new questions.

What makes much of this possible is the Atlas.mask method, which takes in a simple masking function (including custom ones from outside of MaskMyPy) and any keyword arguments for that function. When the method is called, it will apply the masking function to the sensitive points, perform a range of analyses on the resulting masked GeoDataFrame, and then add a ‘candidate’ to a list. A candidate is a simple Python dictionary that contains various statistics describing the mask’s performance (see Table 2), as well as all the information required to regenerate an exact copy of the masked GeoDataFrame. The latter point is significant as it means that the masked GeoDataFrame itself can be discarded from memory after being evaluated, then recreated later if needed. This allows users to iterate over hundreds, or even thousands, of combinations of masks and parameters without running out of memory or disk space. The following code snippet shows how the Atlas can be used to apply donut masking to a set of sensitive points, and the resulting information that is generated:

**Table 2:** A list of the various statistics that can be automatically generated for each candidate

Measure	Definition
*central_drift*	The distance that the mean center of the point pattern has moved
*displacement_min*	The minimum distance that a point was displaced
*displacement_max*	The maximum distance that a point was displaced
*displacement_mean*	The mean distance that points were displaced
*displacement_med*	The median distance that points were displaced
*nnd_min_delta*	The difference between the minimum nearest-neighbour distance of the original point pattern and the masked point pattern
*nnd_max_delta*	The difference between the maximum nearest-neighbour distance of the original point pattern and the masked point pattern
*nnd_mean_delta*	The difference between the mean nearest-neighbour distance of the original point pattern and the masked point pattern
*ripley_rmse*	The root-mean-square error between the Ripley’s K-test results of original and masked points
*k_min*	The minimum k-anonymity that was achieved across all points
*k_max*	The maximum k-anonymity that was achieved across all points
*k_mean*	The mean k-anonymity that was achieved across all points
*k_med*	The median k-anonymity that was achieved across all points
*k_satisfaction_5*	The percent of points that achieved 5-anonymity or greater
*k_satisfaction_25*	The percent of points that achieved 25-anonymity or greater
*k_satisfaction_50*	The percent of points that achieved 50-anonymity or greater
*execution_time*	The amount of time that the masking function took to execute
*memory_peak_mb*	The maximum amount of additional memory that the masking function itself used


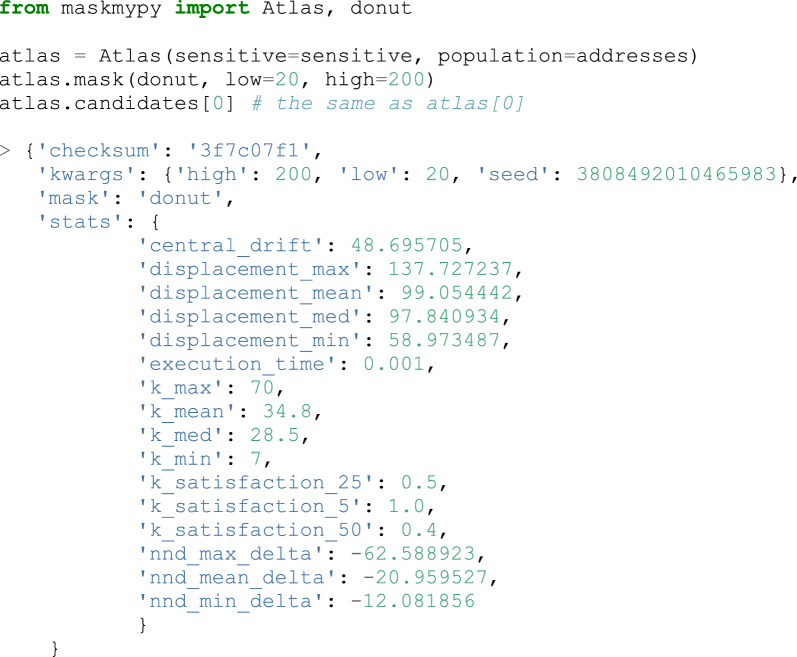


Importantly, the Atlas also helps users find the candidates that meet their criteria. For instance, if the above Atlas contained many candidates, a user can remove any candidates with a minimum k-anonymity below 5, then sort by the median displacement distance using prune and sort methods. Finally, they can export a table (specifically, a Pandas DataFrame, shown in Table 3) of all the remaining candidates:

**Table 3:** A DataFrame generated by the Atlas, illustrating how candidates can be filtered, sorted, and exported based on various statistics. Note that the DataFrame has been significantly truncated for brevity

Mask	Low	High	Displacement_mean	k_min
Donut	20	200	130.608376	8.0
Donut	50	500	251.489335	20.0
Donut	100	1000	518.311502	57.0


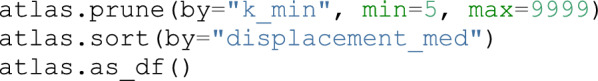


We believe that this degree of ease-of-iteration not only enables users to better protect their data, but also allows scholars researching geographic masks to begin to ask *new questions* about the masks they are developing. The next section exemplifies this by using MaskMyPy to explore mask *variability*.

## Methodology

### Case study: how variable are geographic masks?

Studies that present new geographic masks often follow a general pattern: describe a newly developed mask, then compare it to other masks using a set of test data, a small number of parameters for each mask (e.g. varying the maximum displacement distance), which are each executed only once. However, randomization is a critical component of *most* masks, introducing the possibility that the mask performed well in that particular execution, but may not perform as well in other executions. Using MaskMyPy, particularly the Atlas, we present a short case study on mask consistency, and examine how randomization and dataset size can affect privacy protection.

### Data

The primary dataset used for this case study is a set of synthetically generated points in Vancouver, Canada, which was originally created in [10]. Synthetic datasets are often used in geographic masking research due to obvious privacy risks of using actual sensitive data [6, 8, 28]. More specifically, this dataset includes 7206 points, with clusters deliberately distributed across areas of varying population density. Further details as to the exact generation process can be found in [10]. To test how the size of a dataset affects mask variability, we randomly sampled points from this dataset to create five smaller datasets of 50, 100, 500, 1000, and 5000 points. Address data is also required for both privacy calculations and the location swapping mask, and was obtained from [29].

## Methods

Using MaskMyPy’s Atlas feature, donut masking, location swapping, and street masking were repeated 50 times for each of the five sets of sensitive points. Donut masking and location swapping used minimum and maximum displacement distances of 20 and 200 m, respectively. Street masking used a range of 2 to 9 nodes along the street network, which was selected to produce similar minimum and median displacement distances as donut masking.

To evaluate privacy protection, we selected the k-satisfaction metric described above, which the Atlas automatically calculates at three thresholds of k: 5, 25, and 50. To reiterate, if a dataset contains 100 masked points, and 93 of those points achieved a k-anonymity value greater than or equal to 25, then the k-satisfaction would be 93%. We then created box plots depicting the range of k-satisfaction results across the 50 iterations of each pairing of mask and dataset. This represents the variability of privacy protection provided by the mask.

## Results

**Fig. 3:**
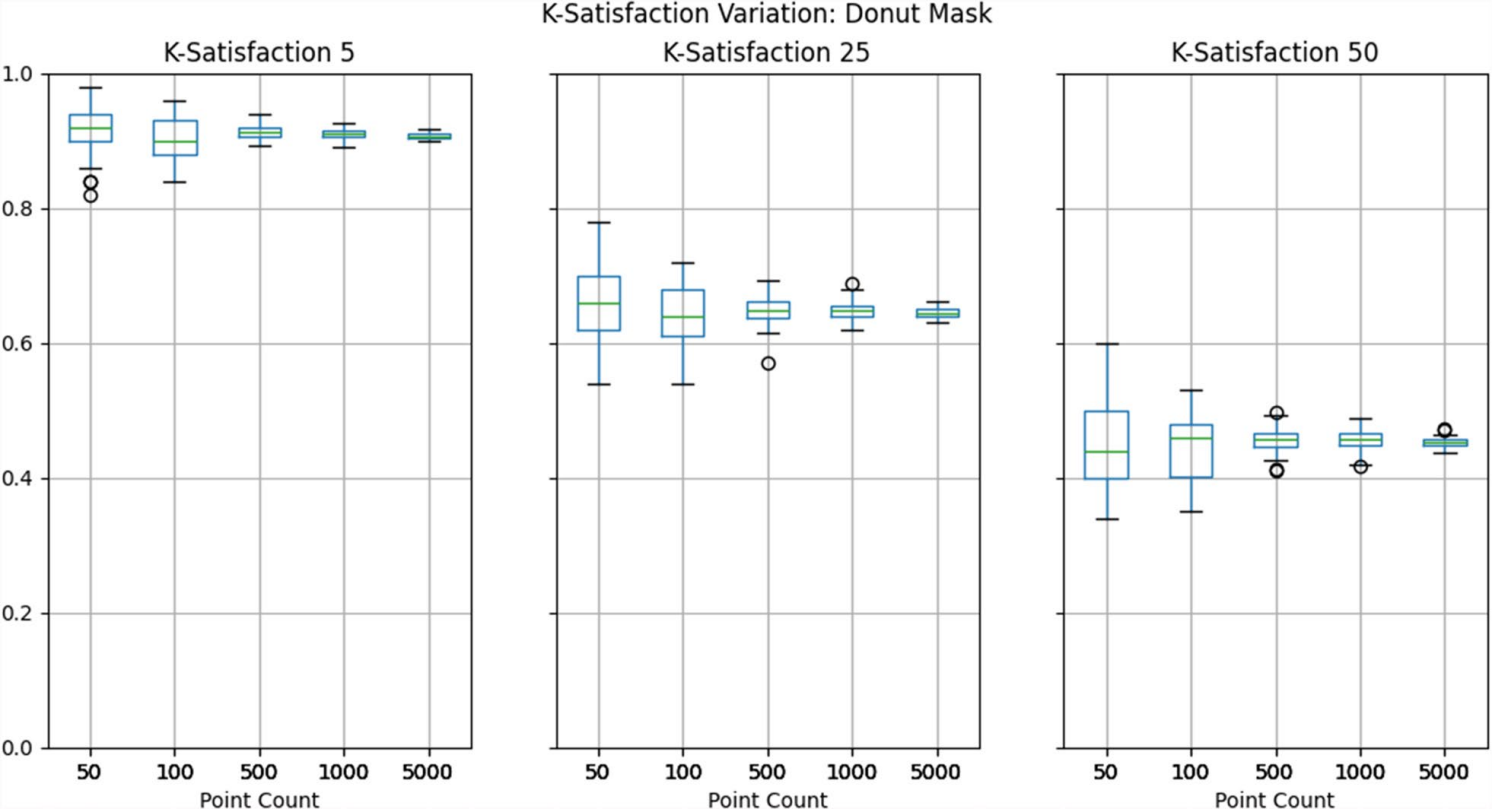
Box-plots depicting the range of k-satisfaction across 50 iterations of donut masking

**Fig. 4:**
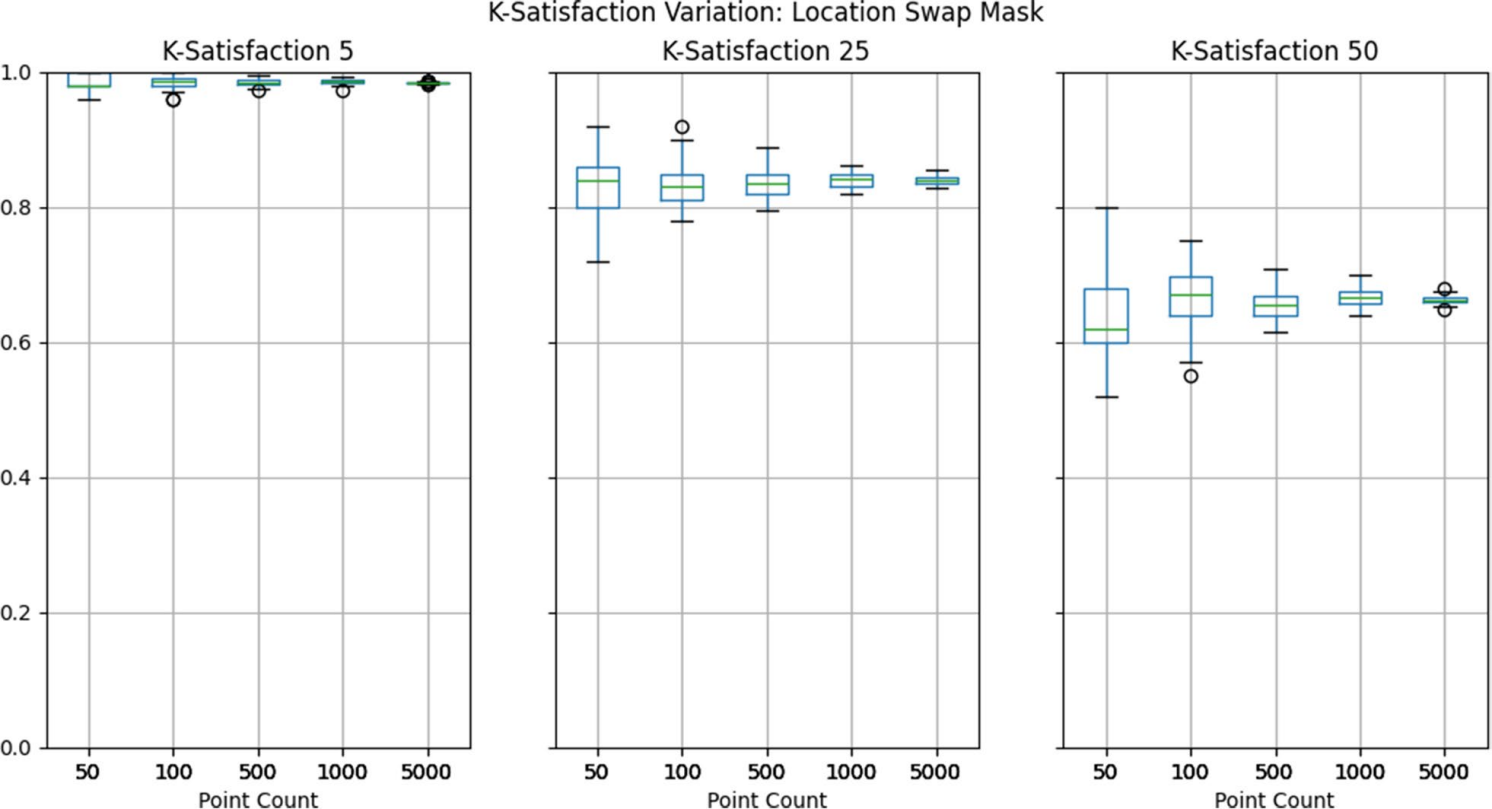
Box-plots depicting the range of k-satisfaction across 50 iterations of location swapping

**Fig. 5:**
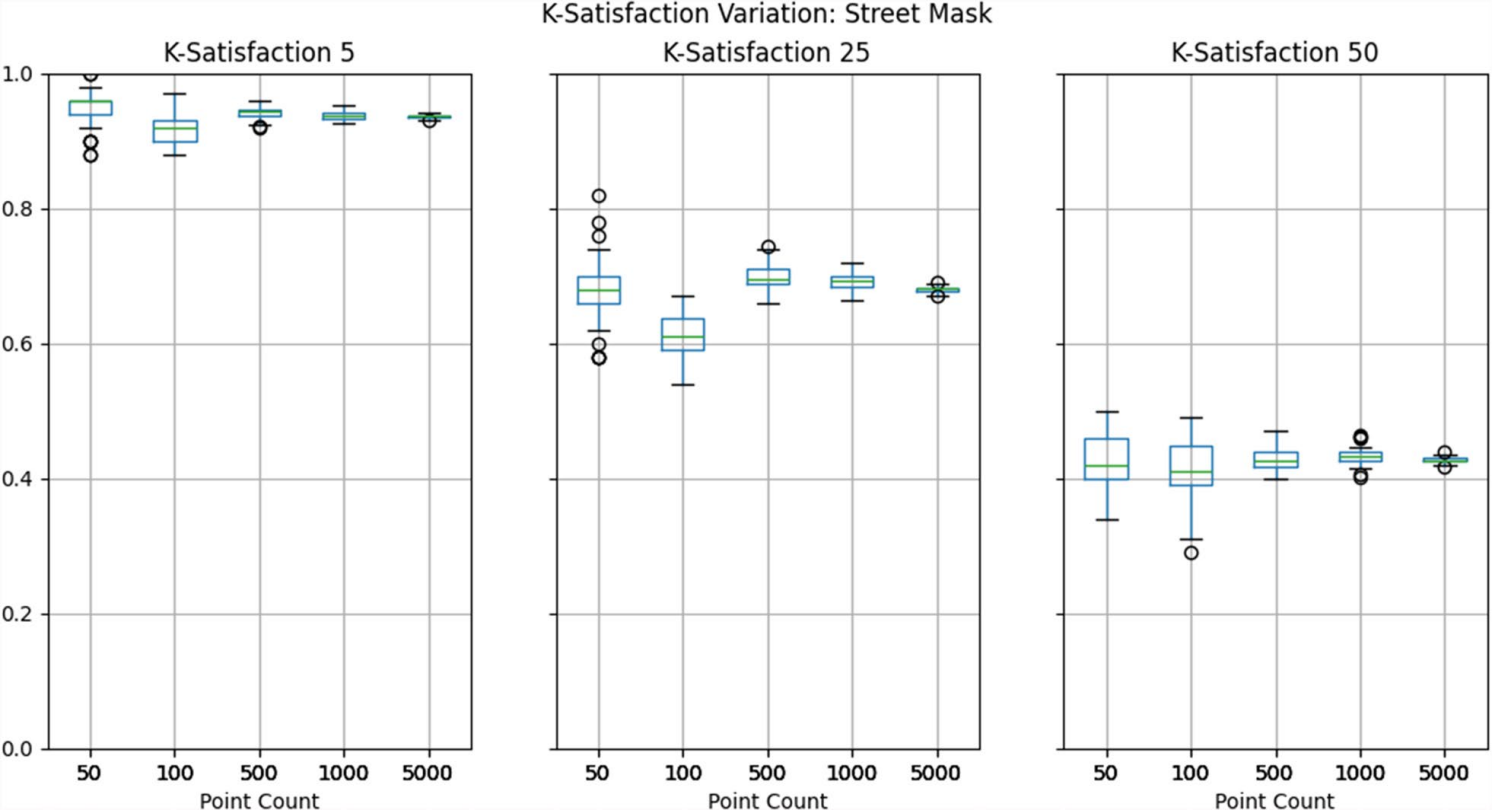
Box-plots depicting the range of k-satisfaction across 50 iterations of street masking

Results show that privacy protection can vary considerably across mask iterations at lower point counts, with higher counts producing more consistent results, as illustrated in Figs. 3, 4, 5 and detailed in Table 4. While this general trend is certainly expected, the range of k-satisfaction performance between different masks should not be overlooked when using fewer than 1000 points. With 500 points and a minimum k of 50, k-satisfaction varied 7–9%, and with 100 points this rose to approximately 20%.

**Table 4:** K-Satisfaction minimums, maximums, and ranges across 50 iterations for each combination of mask and test dataset size

Point Count	Mask	K5 Min	K5 Max	K5 Range	K25 Min	K25 Max	K25 Range	K50 Min	K50 Max	K50 Range
50	Location Swap	0.960	1.000	0.040	0.720	0.920	0.200	0.520	0.800	0.280
50	Donut	0.820	0.980	0.160	0.540	0.780	0.240	0.340	0.600	0.260
50	Street	0.880	1.000	0.120	0.580	0.820	0.240	0.340	0.500	0.160
100	Location Swap	0.960	1.000	0.040	0.780	0.920	0.140	0.550	0.750	0.200
100	Donut	0.840	0.960	0.120	0.540	0.720	0.180	0.350	0.530	0.180
100	Street	0.880	0.970	0.090	0.540	0.670	0.130	0.290	0.490	0.200
500	Location Swap	0.972	0.994	0.022	0.796	0.888	0.092	0.614	0.708	0.094
500	Donut	0.892	0.940	0.048	0.570	0.692	0.122	0.410	0.498	0.088
500	Street	0.920	0.960	0.040	0.660	0.744	0.084	0.400	0.470	0.070
1000	Location Swap	0.973	0.992	0.019	0.820	0.861	0.041	0.640	0.700	0.060
1000	Donut	0.890	0.925	0.035	0.620	0.688	0.068	0.418	0.488	0.070
1000	Street	0.925	0.953	0.028	0.663	0.719	0.056	0.402	0.464	0.062
5000	Location Swap	0.981	0.987	0.006	0.828	0.855	0.027	0.648	0.679	0.031
5000	Donut	0.899	0.917	0.018	0.630	0.662	0.032	0.438	0.472	0.034
5000	Street	0.930	0.942	0.012	0.670	0.691	0.021	0.417	0.439	0.022

Put into context, this means that if two identical masks were tested against each other, one could perform 20% better than the other, despite the only difference being due to randomization. While 20% is at the extreme of this particular test, in practice it is likely that even an 7% difference in k-satisfaction performance could lead one to conclude that one mask is ‘superior’.

It must be noted that this case study was not designed to compare each of the three masks relative to one another, as only a *single set of parameters* was used for each mask; tuning these parameters would likely lead to very different results. Rather, this analysis is meant to illustrate how varied masks can perform due to the randomization that underpins many of them. Indeed, researchers comparing masks should consider the potential for such variation in their study design, whether this be by using larger dataset sizes or multiple iterations of each set of masking parameters. Given that masks may be used by end users on both small and large datasets, it is likely wise to use both approaches side by side to ensure that the mask is performant across a variety of data.

## Discussion & conclusion

Geographic masking is a small but important niche within GIScience. Huge volumes of spatial data are kept behind closed doors in the interest of privacy, but geographic masks offer the potential to open some of this data up to health researchers. Simultaneously, sensitive health data that *should* be protected with geographic masks is often published for the world to see, a practice that occurs far too often and that may (and perhaps, *should*) erode public trust [1, 2]. Finally, there is data that *is* protected before being released, but is based on areal aggregation, which can limit the usefulness of the data by impeding cluster detection [3]. MaskMyPy seeks to tackle each of these cases by removing the friction of robust geographic masking, and by fortifying research in the space.

Of course, it is impossible to make masking entirely friction-less. Users still must understand some of the fundamental concepts that underlie geographic masks, such as a mask’s overall mechanism for displacing points, how k-anonymity relates to privacy, and how information loss may be measured and represented. Tooling alone cannot solve these issues. But just as a user need not be an expert in computational geometry to understand the value that a ‘union’ or ‘intersect’ tool in GIS software may provide them, they need not be experts in geographic masking to get value from masking tools. Furthermore, tools can themselves work to educate users of underlying concepts; there is a reason GIS courses start by having students experiment and play with data and tools before unpacking how those tools work. In this way, we hope that MaskMyPy provides tools for researchers to play with, learn from, and ultimately incorporate into their workflows such that privacy is better protected while rich geospatial datasets are simultaneously opened up to the community.

Moreover, implementation matters, and MaskMyPy’s analysis tools help more advanced users interrogate the masks they implement. This applies not just to novel masks, but existing masks as well, which often lack publicly accessible implementations that users can reference, if not use directly. Indeed, the smallest decisions made when codifying methods can, whether on their own or in aggregate, produce significantly different results. For instance, there are a number of ways one can perform a mask as simple as random perturbation. In one method, a buffer is created around a given sensitive point, then a new point is randomly sampled within the buffer, producing the masked point. However, this method is much slower than just randomly selecting a distance and direction, and translating the point’s geometry accordingly. While this may seem like a simple optimization, the buffer-based approach fundamentally distributes points differently, with displacement distances being biased more towards the outside of the buffer area (where there is more area). The translation approach, on the other hand, produces a far more uniform distribution of displacement distances. This is only one example of myriad decisions made when programming that rapidly branch out into often unexpected results.

However, compounding these issues is that modern software development often heavily relies on third party libraries, which themselves can suffer the same problems. Much like science more broadly, we build on the shoulders of giants, and in doing so inherit many of their idiosyncrasies, whether we know it or not. Of course, this applies to MaskMyPy itself, and anything built on top of it. Comprehensive software testing can help to mitigate this problem, but is not a silver bullet. Software testing is difficult; it can be hard to know *what* to test, never mind how to *meaningfully* test it. This is not to argue against testing, but rather to underscore the importance of open source software in academic research. In this case, MaskMyPy provides masks that can act as reference implementations, and that other researchers can contribute to, whether that be by adding new masks, improving tests, or finding bugs. As such, we would encourage other researchers to not just use MaskMyPy’s masks and analysis tools when developing new masks, but to contribute these masks back into MaskMyPy. Over time, we hope to build a library of masks that the geomasking community can leverage and build around. To join this effort, visit https://github.com/TheTinHat/MaskMyPy.

## Data Availability

The synthetic datasets analysed during this study are available at https://osf.io/6uqkv. MaskMyPy is available at https://github.com/TheTinHat/MaskMyPy.
